# Integrated care for people with multimorbidity into elective surgical pathways: mixed-methods co-design study

**DOI:** 10.1093/bjs/znaf246

**Published:** 2025-11-12

**Authors:** Sivesh K Kamarajah, Jugdeep Dhesi, Kamlesh Khunti, Krishnarajah Nirantharakumar, Paul Cockwell, Clare Hughes, Paul Stern, Joyce Yeung, Dion G Morton, Aneel A Bhangu, Shalini Ahuja

**Affiliations:** Department of Applied Health Sciences, School of Health Sciences, College of Medicine and Health, University of Birmingham, Birmingham, UK; NIHR Global Health Research Unit on Global Surgery, University of Birmingham, Birmingham, UK; Department of Population Health Sciences, Faculty of Life Sciences and Medicine, Kings College London, London, UK; Department of Health and Ageing, Guy’s and St Thomas’ NHS Foundation Trust, London, UK; Diabetes Research Centre, Leicester General Hospital, University of Leicester, Leicester, UK; Department of Population Health Sciences, Faculty of Life Sciences and Medicine, Kings College London, London, UK; Department of Older People Medicine, University Hospitals Birmingham NHS Foundation Trust, Birmingham, UK; Department of Older People Medicine, University Hospitals Birmingham NHS Foundation Trust, Birmingham, UK; Midlands Cardiovascular and Respiratory Clinical Network, Birmingham, NHS England, UK; Warwick Clinical Trials Unit, Warwick Medical School, University of Warwick, Coventry, UK; Department of Applied Health Sciences, School of Health Sciences, College of Medicine and Health, University of Birmingham, Birmingham, UK; NIHR Global Health Research Unit on Global Surgery, University of Birmingham, Birmingham, UK; Department of Applied Health Sciences, School of Health Sciences, College of Medicine and Health, University of Birmingham, Birmingham, UK; NIHR Global Health Research Unit on Global Surgery, University of Birmingham, Birmingham, UK; Centre for Implementation Science, Health Services and Population Research Department, Institute of Psychiatry, Psychology and Neurosciences, King's College London, London, UK

## Abstract

**Background:**

People with multiple long-term conditions (MLTC) commonly undergo elective surgery, yet current pathways remain poorly equipped to meet their complex needs. These pathways present a unique, time-sensitive opportunity to act. The aim of this study was to co-design a feasible intervention that integrates MLTC care into surgical pathways.

**Methods:**

This was a theory-informed mixed-methods co-design study (informed by the National Institute for Health and Care Research (NIHR)/Medical Research Council (MRC) complex intervention framework). Phase 1 involved contextual analysis of current UK pathways (pathway mapping, policy/guideline scan, and national survey) and phase 2 involved multidisciplinary stakeholder workshops to develop a Theory of Change.

**Results:**

In phase 1, pathway mapping identified variation and delayed preassessment, resulting in a limited window to optimize chronic diseases. The scoping review found no UK guidance integrating MLTC into surgical pathways. In the survey (73 responses, 51 National Health Service (NHS) Trusts), few services screened at listing and structured pathways were uncommon. Only one-in-ten hospitals had an MLTC-specific care pathway for elective surgical patients, primarily focusing on diabetes or anaemia management. In phase 2, 21 stakeholders agreed upon a pragmatic intervention prioritized on four domains (diabetes, hypertension, weight management, and smoking cessation), with five intervention components: surgeon-led checklist-based early identification at listing; automated referral to primary care/specialist services; patient-activation materials; optimization during waiting time; and structured discharge communication.

**Conclusion:**

This study presents a co-designed model that shifts MLTC care upstream to the point of listing, offering the potential to improve short- and long-term health.

## Teaser

This study presents a co-designed model that shifts multiple long-term condition (MLTC) care upstream to the point of listing. Although this model offers the potential to improve short- and long-term health, closing the knowledge gap on its clinical effectiveness is required before broader implementation into the National Health Service (NHS).

## Introduction

In the UK, approximately 14 million people, a quarter of the population, currently live with two or more multiple long-term conditions (MLTC; multimorbidity)^1^, a figure projected to double by 2045^2^. Similar trends are observed globally, particularly in developed countries. Despite this growing burden, hospital-based care systems remain predominately structured around single-disease models^3^, posing significant challenges in addressing the complex, often age-related needs of this population. Evidence indicates that patients with MLTC who access secondary care report reduced quality of life, largely due to fragmented services that struggle to provide holistic, patient-centred care^4^. While primary prevention continues to be led by community and primary care services, hospitals now play an increasingly important role in delivering both primary and secondary prevention.

Elective surgery and perioperative care are among the most resource-intensive and modifiable healthcare pathways for patients with MLTC^5^. At present, more than 313 million surgical procedures have been performed worldwide^6,7^, with up to 7 million performed in the UK alone. One-in-two patients undergoing abdominal surgery have MLTC^8^, associated with twice the risk of postoperative complications^8^, prolonged functional decline, and accelerated frailty trajectories; structured pathways provide a unique opportunity to address a chronic disease throughout its course to improve short- and long-term outcomes for patients. Aligned with policies such as ‘Making Every Contact Count’^9^ and the ‘NHS Long Term Plan’^10^ (where NHS stands for National Health Service), the surgical waiting interval should be leveraged not only for treatment but also for proactive prevention. Despite this, for most services in the UK that screen patients, optimization and integration with primary care are variable. Patients often wait weeks or months between listing and surgery, but the system rarely uses that time. The practical problem is two-fold: early identification at the point of surgical listing is uncommon; and continuity across organizational boundaries is unclear. An integrated approach to long-term condition management within surgical pathways can help preserve function, slow decline, and promote healthy longevity. However, there is limited empirical guidance on how to operationalize MLTC-focused surgical care models^11^ .

To bridge this knowledge gap, the aim of this theory-informed mixed-methods co-design study, was to develop an intervention that addresses patients with MLTC undergoing elective surgery in the UK informed by the National Institute for Health and Care Research (NIHR)/Medical Research Council (MRC) complex intervention framework^12^. A mixed-methods approach was used because no single source could define both the problem and a workable solution; pathway mapping and a targeted policy/guideline scan were used to describe current practice and gaps, a national survey was used to gauge reach and variation, and stakeholder workshops were used to specify a testable model through Theory of Change (ToC). Co-design refers to equal partnerships between patients and professionals, to shape what is delivered and how it is delivered^13^, while a mixed-methods strategy combines qualitative and quantitative approaches to explain both what happens and why. Complex interventions consist of several interacting parts and their effects depend on local context; ToC is a step-by-step map that explains how the components within the complex intervention link to short-term milestones and longer-term outcomes. By identifying key challenges and enablers, and integrating theory-informed solutions, this study lays the foundation for future trials aimed at improving long-term postoperative health outcomes for individuals with MLTC.

## Methods

### Study design

**Fig. 1:**
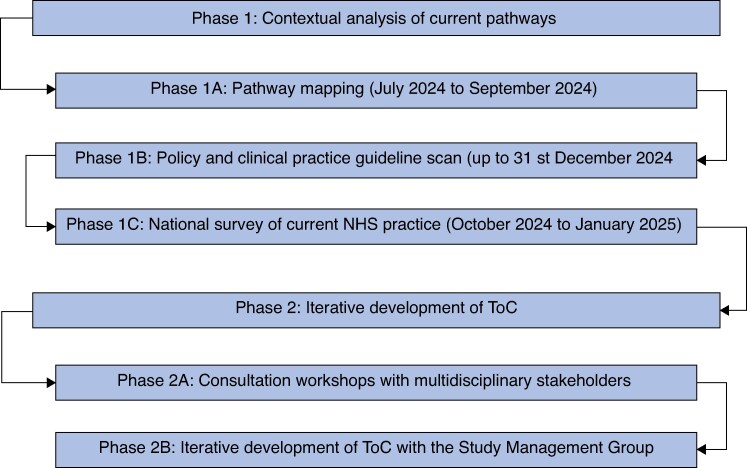
Overview of the mixed-methods study design NHS, National Health Service; ToC, Theory of Change.

This theory-informed mixed-methods co-design study was conducted in two sequential phases (*Fig. 1*): phase 1—contextual analysis of existing surgical pathways; and phase 2—triangulation of findings from phase 1 to co-design and validate a complex intervention using ToC workshops. The co-design process was informed by the NIHR/MRC complex intervention framework^12^ and is reported according to the guidance for the reporting of intervention development (GUIDED) recommendations (*Table S1*)^14^. The final co-designed complex intervention is reported according to the template for intervention description and replication (TIDieR) checklist^15^. This study was reviewed and approved by the University of Birmingham research ethics committee (ERN_2804-Jun2024).

### Phase 1: contextual analysis of current pathways

#### Phase 1A: pathway mapping

From July to September 2024, 12 one-on-one pathway-mapping interviews (up to 60 min) were conducted with purposively sampled NHS stakeholders (surgeons, anaesthetists/perioperative physicians, geriatricians, specialist nurses, pharmacists, primary care clinicians, and operational managers) across teaching and district general hospitals. Each session was facilitated by two researchers trained in human-centred design, employing a paper-based journey-mapping board pre-populated with key anchor points: primary care referral, preoperative assessment, day of surgery, inpatient recovery, discharge, and post-discharge follow-up. Participants annotated the journey with notes indicating actions and information flows. Artefacts (annotated maps, sticky-note clusters, facilitator field notes) were photographed, digitized in Microsoft PowerPoint on the same day, and version controlled to create an evolving composite map. A framework-analysis approach guided coding of case notes, using a priori categories such as touch points, actors, information exchanges, delays, integration gaps, and inductive codes for emerging bottlenecks. Two analysts coded the data independently using a shared codebook; discrepancies were discussed and resolved by consensus, with a third reviewer available if needed. A κ statistic was not calculated, given the developmental focus of this work, but agreement was monitored qualitatively and documented throughout. Member checking took place at the start of each subsequent workshop^16^; the composite pathway was presented back to participants for confirmation or correction; all changes were logged. The Study Management Group (multispecialty) reviewed the final composite map for face validity before use in phase 2.

#### Phase 1B: policy and clinical practice guideline scan

This phase aimed to identify UK policy and guideline recommendations relevant to integrating MLTC into elective surgical pathways. To do so, the public repositories and websites of the National Institute for Health and Care Excellence (NICE), NHS England, the Centre for Perioperative Care (CPOC), relevant Royal Colleges, and professional societies (from inception to 31 December 2024) were searched and reference lists and expert suggestions from the Study Management Group were checked. UK national policies, clinical guidelines, or consensus statements with perioperative relevance to MLTC or its common domains (for example diabetes, hypertension, smoking) in adults undergoing elective surgery were included. The following were excluded: single-centre protocols, non-UK policies, and documents without perioperative recommendations. Two reviewers independently screened titles or text and extracted source, scope, target population, and actionable perioperative recommendations; disagreements were resolved by discussion. Recommendations were summarized narratively and tabulated by domain; no meta-analysis was planned.

#### Phase 1C: national survey of practice

To better understand real-world perioperative care for patients with MLTC and address gaps identified in phase 1A (pathway mapping) and phase 1B (guidelines), a national survey targeting healthcare professionals involved in elective surgical care across the UK was designed. This survey aimed to: describe current practices in assessing and optimizing long-term conditions (for example diabetes, hypertension, smoking cessation) before elective surgery; and identify barriers and facilitators to delivering integrated perioperative care for patients with MLTC. Developed in collaboration with the Study Management Group, including surgeons, anaesthetists, perioperative physicians, nurses, primary care clinicians, and patient representatives, the survey questions were structured around themes emerging from phase 1A and phase 1B (for example screening, optimization, communication, handover). To ensure breadth and depth in responses, a mixture of closed multiple-choice items (describing service structures and practices) and open-ended questions (capturing perceived barriers and innovative approaches) were incorporated. Barriers and facilitators were analysed thematically using the Consolidated Framework for Implementation Research (CFIR)^17,18^, mapping responses onto relevant domains and constructs. Responses were systemically reviewed, with emergent themes grouped inductively and deductively, guided by the updated CFIR 2.0 framework, including ‘intervention characteristics’, ‘inner and outer setting’, ‘individuals’, and ‘implementation processes’. To further refine the assessment, ‘implementation outcomes’ such as feasibility and acceptability were incorporated. Data were triangulated across key perioperative components (for example screening, medication management, care planning, prehabilitation), supported by illustrative quotes to reinforce key findings. Before launch, the survey underwent pilot testing with five clinicians in various roles to ensure clarity and usability. The survey was distributed electronically using Research Electronic Data Capture (REDCap) software and disseminated through national research networks focused on surgical care. A core sample of 126 clinicians from 65 NHS Trusts within the authors’ existing research network were invited directly by e-mail. To broaden reach, the survey link was also disseminated via professional organizations (the Royal College of Surgeons, the Association of Anaesthetists, the COPC, and the perioperative section of the British Geriatrics Society) and through snowball sampling, where respondents were asked to forward the link within their hospitals. Participation was anonymous, with consent implied through response submission. The survey remained open between 9 September 2024 and 15 November 2024 (see the *Supplementary material* for the complete survey).

### Phase 2: iterative development of ToC

A ToC is defined as ‘a theory of how and why an initiative works which can be empirically tested by measuring indicators for every expected step on the hypothesized causal pathway to impact’^19^. A detailed description of the methodology is available in the *Supplementary material*. To develop the ToC, key stakeholders were convened from three hospitals, each participating in a dedicated workshop. These workshops followed a structured format, including an introduction of the project and the ToC framework, discussion of the significance of integrated surgical pathways for patients with MLTC, and a mapping exercise using structured group discussions and small group activities. Unlike conventional workshops that primarily gather views and opinions, ToC workshops are distinguished by their tangible output: a ToC map, developed collaboratively and agreed upon by stakeholders. During each ToC workshop, stakeholders first defined the impact and long-term outcome of an integrated approach to surgical care for patients with MLTC. They then worked ‘backwards’, systemically identifying all necessary preconditions, using visual aids, such as whiteboard mapping. This process was repeated iteratively until consensus was reached on both the content and chronological sequencing of the preconditions. Once agreement was established, stakeholders formulated intervention components and activities required to achieve the identified preconditions. Facilitators then presented the draft ToC map in poster format, ensuring all participants shared a common understanding of the causal relationships outlined in the map. Throughout the workshops, participants were encouraged to reflect on their reasoning behind each causal relationship, specify why specific interventions were necessary for achieving desired outcomes, and explicitly articulate assumptions about potential implementation barriers within local healthcare contexts.

After the completion of all workshops, a draft ToC map was developed and subsequently reviewed in two meetings with the Study Management Group. These meetings focused on refining the formulation of the preconditions, assessing their causal relationships, and finalizing the intervention components within the ToC framework. During this phase, a ToC expert (S.A.) ensured methodological accuracy, verifying that terminology and causal pathways were consistent. The finalized map was further validated against relevant literature recommended by the Study Management Group and assessed based on the four key attributes of a robust ToC (plausibility, feasibility, meaningfulness, and testability). To maintain a comprehensive record, the first author transcribed video and audio recordings of the workshops (for which participants provided verbal consent) and captured photographs of the ToC map at the conclusion of each session. Key points raised by stakeholders, particularly those deemed critical to implementation, were incorporated into the final map.

### Patient and public involvement

UK NIHR standards were followed for patient and public involvement. One patient partner participated in each study phase. Patient partners were recruited from the authors’ hospital patient advisory network and had lived experience of MLTC and elective surgery. Patient partners contributed to: overall study design (refining aims and scope); prioritization (judging importance and feasibility of candidate domains); specification (language and format of patient-facing materials and discharge content); and interpretation (sense checking pathway maps and the ToC). Notes of all patient contributions and resulting changes were logged and verified with the partner at the next session.

## Results

### Phase 1: contextual analysis of current pathways

#### Phase 1A: pathway mapping

**Fig. 2:**
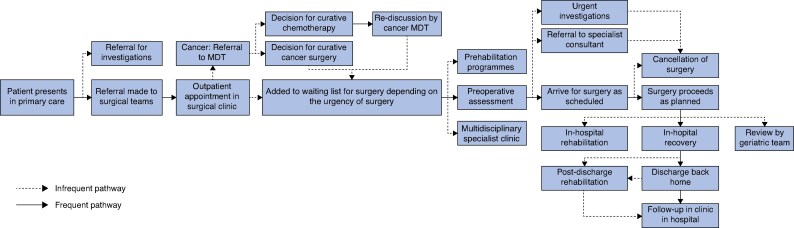
Composite perioperative pathway for elective surgery across the NHS This pathway is derived from one-to-one mapping interviews. Core steps (present in most sites) form the main line of the pathway; site-variant options are shown as labelled branches where practices diverged (for example timing/locus of preassessment, referral triggers, discharge letter content). The map was member checked with participants and underwent face-validity review by the Study Management Group before phase 2. NHS, National Health Service; MDT, multidisciplinary team.

Between July and September 2024, 12 site-specific pathway-mapping sessions were conducted with 20 multidisciplinary stakeholders (including 4 surgeons, 3 perioperative physicians, 4 geriatricians, 2 nurses and allied health professionals, 1 primary care clinician, and 1 patient representative). A detailed description of the expert stakeholders is provided in *Table S3*. Using a standardized journey-mapping board (that is referral, listing, preassessment, optimization during waiting, day of surgery, discharge, and postoperative follow-up), a composite pathway describing the patient journey was produced (*Fig. 2*). Four recurring structural bottlenecks were identified. First, limited preoperative optimization; assessments were typically scheduled only 2 weeks before surgery, restricting the opportunity for intervention in high-risk patients. Second, inadequate chronic disease management; conditions such as diabetes, hypertension, and anaemia were poorly optimized before hospital admission, leading to delays or cancellations. Third, fragmented information systems; clinicians manually reconciled medication and investigation data across disconnected records, increasing inefficiencies. Fourth, delayed lifestyle-risk interventions; smoking cessation efforts were initiated too late in the pathway, despite many patients being listed for surgery months in advance. Across sites, consistent gaps in core steps were identified, but there were notable site-variant options with regard to: timing and locus of preassessment (surgical clinic *versus* perioperative medicine clinic); source of referrals (initiated by a surgeon *versus* initiated by a specialist nurse); and content of discharge letters (presence/absence of explicit MLTC actions and targets). These differences were preserved in the composite pathway as clearly labelled optional branches, while core steps were defined by majority practice. Member checking confirmed the composite pathway reflected local realities; suggested edits focused on labelling and sequencing rather than adding new steps. To address these deficiencies, stakeholders identified four high-impact solutions through consensus voting: routine MLTC screening at the point of surgical listing; automated digital referrals to primary care or disease-specific services; a shared electronic care plan accessible across healthcare settings: and a named care coordinator for continuity throughout the perioperative interval.

#### Phase 1B: policy and clinical practice guideline scan

Ten documents were identified, but only two explicitly acknowledged the growing MLTC population; none provided recommendations for surgical pathways. Most guidelines focused on condition-specific management, lacking integration across co-morbidities (*Table S4*). While some addressed prevention strategies, none established structured perioperative pathways, particularly for smoking cessation. Furthermore, the majority of recommendations were based on expert consensus rather than graded evidence. Mapping these guidelines onto current surgical pathways revealed three significant gaps. First, an absence of guidance linking chronic disease screening to surgical listing decisions—assessments remain limited to late-stage preoperative clinics. Second, a lack of protocols for primary care referrals triggered by positive screening results. Finally, discharge summaries focus primarily on procedural details, neglecting ongoing optimization needs.

#### Phase 1C: national survey of practice

**Fig. 3:**
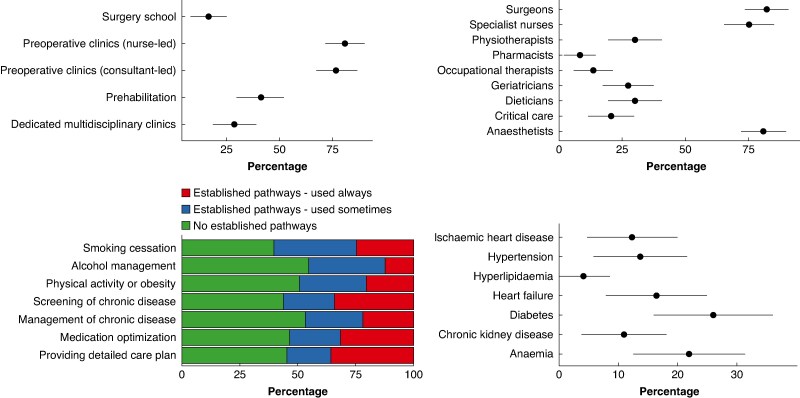
Current national perioperative care practice across the NHS. They show the various types of pre-operative assessment models available, the individuals involved in these services, the interventions performed in them and the type of chronic disease pathways available. NHS, National Health Service.

From 126 participants, 73 responses were from clinicians across 51 NHS Trusts, representing all four UK nations (response rate of 57.9% (73 of 126)) (*Fig. 3*). Respondents spanned various specialties, including surgery (48 respondents (65.7%)), perioperative medicine (14 respondents (19.2%)), geriatrics (10 respondents (13.7%)), and allied healthcare (1 respondent (1.4%)). The predominant preoperative assessment models included nurse-led clinics (80.8% (95% c.i. 71.8% to 89.9%)), consultant-led clinics (76.7% (95% c.i. 67.0% to 86.4%)), prehabilitation (41.1% (95% c.i. 29.8% to 52.4%)), dedicated multidisciplinary clinics (28.8% (95% c.i. 18.4% to 39.2%)), and surgery school (16.4% (95% c.i. 7.9% to 24.9%)). Despite routine communication with primary care providers, dedicated MLTC-specific protocols or pathways remained scarce. Only 25 (34.2%) of respondents reported routinely screening all surgical patients for chronic conditions such as diabetes and hypertension at the time of referral or listing. Established pathways were particularly limited for smoking cessation (18 respondents (24.7%)), alcohol management (9 respondents (12.3%)), and physical activity or obesity (15 respondents (20.5%)).

Thematic analysis using the CFIR framework mapped barriers and facilitators across intervention components (screening, medication management, weight management, smoking or alcohol cessation, and care planning). Primary barriers included workforce shortages (27 respondents), particularly among specialist nurses and anaesthetists, exacerbated by underfunding and lack of commissioning support (‘Inner setting: Available resources’). Fragmentation in communication (18 respondents), between preassessment clinics and cancer nurse specialists, further impeded care coordination (‘Inner setting: Networks and communication’). Facilitators included nurse-led or multidisciplinary prehabilitation models (21 respondents), structured care planning and education resources (11 respondents), and digital or community-based delivery mechanisms improving access despite geographical constraints (‘Intervention characteristics: Design quality and packaging’). Participants emphasized optimization strategies such as early triage, embedded at the time of surgical listing, supported by electronic prompts and automated referral pathways (‘Process: Engaging’ and ‘Process: Executing’). Patient engagement was improved when care plans were personalized and initiated early (12 respondents). Conversely, passive referrals, inconsistent timelines, and unclear role ownership reduced intervention feasibility and acceptability (‘Implementation outcomes’). External constraints such as organizational siloes, such as surgeon-only referral authority and regional policy variations, limited inter-service collaboration (‘Outer setting: Cosmopolitanism’ and ‘Outer setting: External policy and incentives’). A summary of the barriers and facilitators is presented in *Table 1*, with a detailed breakdown by intervention component in *Tables S5–S11*.

**Table 1:** Key barriers and facilitators across intervention components for improving pathways for people with MLTC

Construct	Theme	Barriers	Facilitators
**Inner setting**			
Networks and communication	Limited coordination between CNS, preoperative, and surgical teams	‘Care plan provided by CNS but disjointed from preassessment team.’ (*n* = 18)	‘Coordination via the POM nurse and MDT involvement works well.’ (*n* = 14)
Available resources	Workforce shortages and funding constraints undermine delivery	‘Nursing staff is limited. So, challenging during their leaves.’ (*n* = 27)	‘Dedicated perioperative teams of anaesthetists, physicians and CNSs.’ (*n* = 15)
Compatibility	Integration of new roles and services depends on culture and structure	‘Pharmacy feel it’s not their responsibility. No funding for this.’ (*n* = 7)	‘Periop physicians embedded in team culture improve delivery.’ (*n* = 9)
**Outer setting**			
Patient needs and resources	Prehabilitation and behaviour change interventions are valued, but inconsistently delivered	‘Cost and logistical requirements; prehab not funded in many areas.’ (*n* = 22)	‘Prehab for cancer is brilliant. It can change patients’ lives.’ (*n* = 21)
External policy and incentives	Local commissioning and service variation affects delivery	‘Online referral depending on which council area the patient lives.’ (*n* = 9)	‘Computerized referral pathways save time and reduce admin.’ (*n* = 7)
External partnerships and care coordination	Primary care capacity and coordination constraints resulting in hospital links delay follow-up and optimization	‘Referral must come from surgeon but is identified by anaesthetist—leads to delays.’ (*n* = 11)	‘Seamless and integrated primary–secondary care EHR.’ (*n* = 6)
**Characteristics of individuals**			
Knowledge and beliefs	Staff uncertain about whose role it is to deliver elements of care	‘Preop assessment feel it’s not their responsibility. No CNS time to support.’ (*n* = 13)	‘Staff willing to engage and advise as long as issue picked up by pathway.’ (*n* = 10)
**Intervention characteristics**			
Complexity	Medication management processes are fragmented and variable	‘No routine review of medications to ensure patients are on the most appropriate medications.’ (*n* = 16)	‘Nurse-led preassessment clinics will issue bespoke advice on DOACs, antihypertensives, and diabetes medication.’ (*n* = 12)
Design quality and packaging	Structured patient education tools support engagement	‘No booklet to describe the entire pathway.’ (*n* = 9)	‘Information leaflets and videos provided by nurse specialists. Also supported by enhanced recovery apps.’ (*n* = 11)
**Process**			
Engaging	Early triage and signposting to support services improves uptake	‘Patients often referred too late to benefit from support services.’ (*n* = 17)	‘Prehabilitation team contact patients from the point of listing. Signposting at first OPD.’ (*n* = 18)
Executing	Protocols function well when embedded and streamlined	‘Referral process is ad hoc and often person-dependent.’ (*n* = 11)	‘Blanket offering of prehab and screening via electronic checklists.’ (*n* = 14)
Reflecting and evaluating	Little feedback collected to refine pathways	‘We don't have any data on the effect of our intervention.’ (*n* = 6)	‘Patients give feedback and care plans are reviewed annually.’ (*n* = 5)
**Implementation outcomes**			
Acceptability	Patients engage more when care is personalized and anticipatory	‘Short time between preop and surgery means patients are overwhelmed and poorly prepared.’ (*n* = 10)	‘Patients issued with health plan from CNS preop including stages of pre/intra/postop.’ (*n* = 12)
Feasibility	Staff find it difficult to operationalize all components	‘Too great a task to assess all patients’ medications with their full history.’ (*n* = 9)	‘Templates and resources support routine use.’ (*n* = 7)
**Health equity**			
Structural determinants	Service access is constrained by geography, commissioning, and NHS hospital engagement	‘Limited capacity in a very small NHS board.’ (*n* = 13)	‘Online and community-delivered prehab allows access despite geography.’ (*n* = 7)

MLTC, multiple long-term conditions; CNS, clinical nurse specialist; POM, perioperative medicine; MDT, multidisciplinary team; EHR, electronic health record; DOACs, direct oral anticoagulants; OPD, outpatient department; NHS, National Health Service.

### Phase 2: iterative development of ToC

Three workshops involving 21 stakeholders (6 surgeons, 4 anaesthetists and perioperative care physicians, 5 geriatricians, 4 nurses and allied health professionals, 1 primary care clinician, and 1 patient representative) reached consensus on the intervention and outcomes. The agreed long-term aim was to extend healthy longevity by reducing surgical complications and preserving independence. This was expressed as a 12-month composite outcome combining improvement in EQ-5D-5L (quality of life) with sustained control of diabetes and hypertension. Both 30-day and 6-month milestones were identified as proximal steps toward this outcome. Stakeholders prioritized four domains (that is smoking cessation, weight management, diabetes, and hypertension) and five core intervention components based on feasibility and potential impact: early identification of patients living with MLTC at the time of surgical listing; patient-activation materials to support engagement and self-management; optimization of MLTC within the perioperative window to enhance surgical readiness; a structured healthcare checklist to standardize perioperative assessments; and improved discharge communication to ensure continuity of care post-surgery. They also highlighted key operational preconditions (checklist fields in clinical systems, clear clinical ownership, accessible patient materials, and feedback mechanisms) and critical assumptions (primary care capacity, digital interoperability, access to optimization services, equity safeguards).

**Fig. 4:**
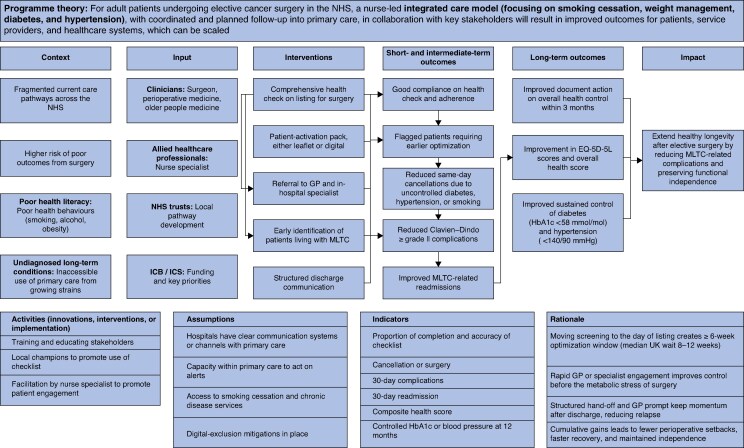
ToC for integrated management of people with MLTC This has been developed through co-design workshops and reviewed by the Study Management Group and multidisciplinary teams in three NHS hospitals. The model is pre-implementation and not yet piloted; it is intended to guide feasibility testing and external validation. Although anaemia was identified in pathway mapping, the final intervention focused on key domains where gaps in long-term perioperative care remain; perioperative anaemia is well supported by existing evidence and pathways. ToC, Theory of Change; MLTC, multiple long-term conditions; NHS, National Health Service; ICB, integrated care board; ICS, integrated care services; GP, general practitioner; HbA1c, haemoglobin A1c.

**Fig. 5:**
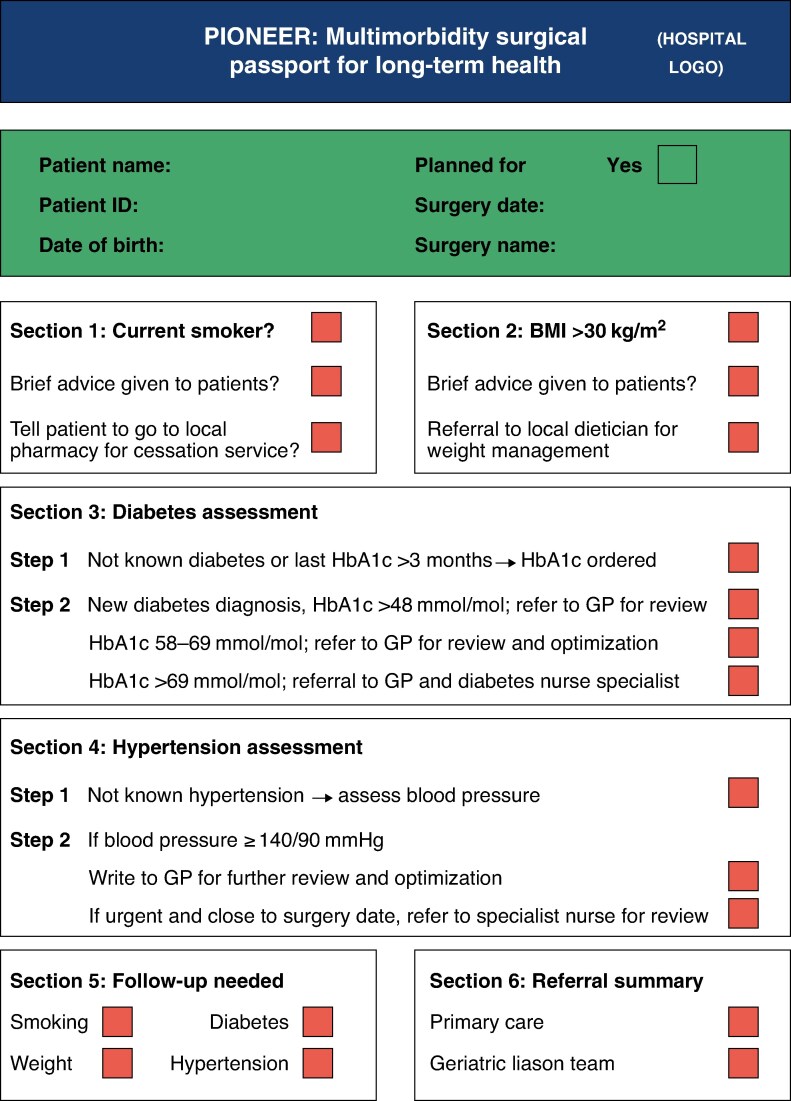
Template checklist for implementation into surgical clinics This template is adaptable based on local pathways across primary and secondary care. HbA1c, haemoglobin A1c; GP, general practitioner.

The final ToC model (*Fig. 4*) illustrates how early identification at surgical listing creates a ≥6-week optimization window, improving perioperative outcomes and supporting post-surgical recovery. A linked measurement set was embedded to facilitate future evaluation, tracking key indicators such as optimization rates, cancellations, readmissions, and long-term health outcomes. Data sources include Trust systems and general practitioner records (*Table S12*). This model now serves as the foundation for pilot testing and economic evaluation of the intervention. A detailed description of the intervention, reported according to the TIDieR guidance, is presented in *Table 2*. An example of the checklist for implementation is presented in *Fig. 5*.

**Table 2:** Description of the co-designed intervention for people with MLTC undergoing elective surgery, according to the TIDieR guidance

Construct	Theme
Name	‘MLTC Surgical Optimization Pathway’; a stakeholder-informed intervention designed to embed chronic disease management and personalized care into elective surgical pathways
Why (rationale)	The intervention addresses missed opportunities for improving outcomes in patients with multimorbidity undergoing elective surgery; it responds to four key system-level deficits identified during pathway mapping and a national survey: Late-stage identification of MLTC Poor optimization of chronic conditions Fragmented care planning and information systems Inadequate discharge continuity
Materials	A package of standardized, scalable components co-designed with clinicians and patients: A digital ‘MLTC Health-Check Checklist’ embedded in electronic patient records Autogenerated referrals to GPs or specialist nurses Patient-facing educational materials (digital and print) including QR-linked resources Structured discharge summaries with optimization follow-up plans
Procedures	The intervention is initiated at the point of surgical listing and includes the following steps: Completion of the MLTC checklist during outpatient surgical appointments Autoreferral of flagged cases to primary care or disease-specific services within 48 h Initiation of optimization (for example medication titration, smoking cessation referral) within 14 days Provision of tailored patient information to support behavioural change Structured discharge plans shared with primary care to ensure postoperative follow-up Complex pharmacotherapy follows local perioperative protocols and specialist clinics; the intervention standardizes when and how referrals are triggered and how plans are handed over at discharge
Providers	Surgeons or surgical nurses complete the MLTC checklist at the listing appointment Primary care clinicians (GPs or disease-specialist nurses) enact optimization plans CNSs and perioperative teams lead patient education and care coordination Pharmacists support medication review and complex risk management through existing local pathways; the intervention ensures referrals to these services are initiated earlier and include medication actions
Modes of delivery	The intervention is delivered through a combination of: Face-to-face consultations at surgical outpatient clinics Digital health tools and printed educational resources for patients
Location (settings)	Initiated in secondary care surgical clinics (hospital outpatient departments), with subsequent actions coordinated across: Primary care settings for follow-up and chronic disease management Community services (for example smoking cessation, diabetes care) Specialist perioperative clinics as required
Dose (schedule and intensity)	The intervention is delivered across three distinct phases: ‘Day of surgical listing’: initial checklist screening and referral ‘0–14 days post-listing’: patient engagement and initiation of optimization strategies ‘Day of surgery to 30 days after surgery’: structured discharge and follow-up
Tailoring	Adaptations occur at multiple levels to ensure contextual relevance: Risk-based tailoring of optimization (for example smoking, diabetes, hypertension) Flexible referral routing based on local service availability Patient education materials adapted to health literacy and digital access needs Implementation strategies adjusted to local EHR and staffing infrastructure
Modifications	Iterative refinements were made during the ToC workshops: Shifted timing of intervention from preassessment to surgical listing Clarified roles for checklist delivery and referral actions Streamlined referral forms and patient-facing content for clarity and usability Integrated digital dashboards to support audit and learning cycles
Planned fidelity assessment	The intervention includes a built-in monitoring framework with the following fidelity indicators: Proportion of patients with completed and accurate checklists Optimization uptake (for example medication titration, smoking cessation) Surgical cancellations due to modifiable risk factors 30-day complication and readmission rates 6- or 12-month control of HbA1c, BP, and EQ-5D-5L outcomes, using primary and secondary care data

Item 12 of TIDieR (actual fidelity) was removed as the intervention has not yet been trialled; fidelity outcomes are currently unknown. Assessment is planned during forthcoming feasibility and pilot testing phases, including evaluation of implementation consistency across sites. MLTC, multiple long-term conditions; TIDieR, template for intervention description and replication; CNSs, clinical nurse specialists; EHR, electronic health record; ToC, Theory of Change; HbA1c, haemoglobin A1c; BP, blood pressure.

## Discussion

Across the UK, approaches to identifying and managing MLTC in surgical pathways vary widely. Preoperative assessment clinics are standard, but often scheduled close to the operation, leaving too little time to optimize chronic disease or support behaviour change such as smoking cessation. Earlier opportunities are routinely missed during the months between referral and surgery. Pathway mapping confirmed the costs of fragmentation, last-minute cancellations, and poorly controlled long-term health conditions, while the policy scan identified no national guidance integrating MLTC into surgical care. In response, this study co-designed a pragmatic framework that uses the waiting interval for proactive optimization and long-term health management. Action is moved to the moment of surgical listing, using a brief checklist with e-referral triggers to make action reliable and embedding structured discharge communication with explicit targets and a named service. Together, these steps create a continuous chain of care between hospital and primary care, turning familiar tasks into a timely, systematic, and connected process.

Over the past decade, there have been advances in surgical pathways. For instance, the orthogeriatric model was associated with improved outcomes for patients undergoing surgery for hip fractures and has been adopted in many countries^20–22^. However, similar models are infrequent in other types of surgery. Prehabilitation programmes have demonstrated benefits in physical capacity and shorter hospital stays, yet a recent network meta-analysis^23^ found that most focus on exercise, nutrition, and psychology, with limited attention to systematic chronic disease screening or optimization. Additionally, these programmes are often resource intensive and challenging to scale within constrained health systems, resulting in limited adoption across the UK^24–26^. In bariatric surgery, structured preoperative preparation, multidisciplinary review, patient education, and long-term follow-up are standard of care^27,28^. The presented model adapts these principles to general elective pathways, but moves the action to earlier, at listing, with planned handover at discharge.

However, no robust studies have yet examined the best approaches to optimizing care for the growing MLTC population requiring elective surgery. Findings from the pathway-mapping workshops and national survey revealed low rates of screening for diabetes or hypertension at listing and only one-in- five hospitals have structured pathways for patients with MLTC. This fragmentation may explain high rates of day-of-surgery cancellations^29^, ranging from 6% to 30% of elective cases across UK hospitals, often due to inadequate surgical preparedness. These late cancellations not only result in wasted operating-theatre time but also expose patients with MLTC to prolonged waiting times, worsened disease control, and increased anxiety. While several complex interventions have been trialled to MLTC care^11^, their evaluations have generally lacked rigour and scope. Reported improvements, such as modest reductions in length of stay or readmissions, have not translated into consistent, long-term benefits or sustained functional independence. In contrast, the intervention proposed in this study takes a fundamentally different approach, shifting the point of intervention upstream to the surgical listing stage. Early identification and management of modifiable risks at this stage aligns with chronic disease management models used in primary care, where early intervention has been shown to improve long-term health outcomes^30,31^. The model does not replace specialist medication pathways. It moves identification and handover to earlier and makes them automatic, then relies on existing local protocols for complex pharmacotherapy (for example anticoagulation/antiplatelet management, insulin adjustment, resistant hypertension, steroid cover). In practice, this means the checklist at listing triggers referral to the locally agreed service and the discharge letter carries a brief medication plan and named contact. During feasibility, it will be documented whether sites have these pathways, how fast they respond, and what capacity constraints exist.

Stakeholders from the ToC workshops prioritized sustained disease control and preserved independence as key outcomes, plus stable glycaemic and blood-pressure control, ensuring long-term disease stability. Traditional short-term metrics such as 30-day complications, while informative, fail to capture the 1-year excess mortality and functional decline documented in MLTC cohorts after major surgery. Evaluating long-term health aligns surgical quality with both the ‘NHS Long Term Plan’^10^ and patient priorities. Composite outcomes such as EQ-5D-5L and days alive and out of hospital are increasingly utilized, but are typically restricted to 90 days. Extending evaluation to 6–12 months aligns with the published core outcome set for MLTC patients^32^ and enables a more comprehensive assessment of sustained recovery. However, measuring long-term outcomes presents challenges, as multiple factors beyond the surgical episode influence results. This reinforces the importance of interventions that embed clear handovers to primary care and facilitate continuity of care after discharge. Emerging evidence from clinical decision support tools in chronic disease management suggests that sustained intervention improves long-term control of conditions such as diabetes and hypertension^33^. By incorporating similar principles into surgical care, the gap between perioperative planning and long-term health maintenance can be bridged.

A key strength of this study is its structured, theory-informed approach, grounded in the NIHR/MRC complex intervention framework. By incorporating triangulated findings from three complementary sources, the credibility and depth are strengthened. Furthermore, the ToC framework enabled the development of a clear logic model, making the intervention both practical to implement and evaluable in future research. However, several limitations should be acknowledged. The policy and clinical practice guideline scan, conducted rapidly, focused solely on national UK guidelines, potentially overlooking local protocols, grey literature, or international innovations through an exhaustive database coverage. Pathway mapping, workshops, and surveys had limited representation, reflecting only a subset of NHS Trusts across the UK. Findings may not fully capture variation in surgical pathways, commissioning models, or workforce availability nationwide. A formal inter-rater reliability coefficient (for example κ) was not calculated; instead, discrepancies were resolved by consensus. Future evaluations will report quantitative agreement statistics. The national survey relied on clinician self-report, introducing possible social desirability bias or overestimation of best practice adherence. Additionally, the survey was also circulated via professional mailing lists and snowball sampling, so the true denominator is larger and the true response rate is lower and cannot be precisely calculated. Nevertheless, responses were geographically widespread and spanned all major professional roles. Voluntary participation also risks response bias. The PPI model used one patient partner per phase, limiting experience diversity; the authors will expand to a standing panel of at least six patient advisors, apply GRIPP2 reporting prospectively, and co-produce patient-facing materials during feasibility testing. Although the ToC has undergone extensive review by the Study Management Group and across three hospitals, it has not yet been piloted or externally validated in routine care. Refinement of the ToC by the Study Management Group may also have introduced consensus bias, although all changes were logged and externally facilitated. Therefore, transferability beyond these settings remains to be tested; it is planned to validate this prospectively by repeating logic-model walk-throughs in additional hospitals, measuring acceptability, adoption, and fidelity, and adapting the model to local context. Finally, the intervention focuses on four modifiable risk factors (diabetes, hypertension, being obese, and smoking), owing to their high prevalence, feasibility for early optimization, and greater implications for long-term health. However, other critical factors such as frailty, polypharmacy, mental health needs, and chronic pain are yet to be incorporated. While anaemia was highlighted during pathway mapping, it was not prioritized because robust evidence and established optimization pathways already exist^34,35^. Further, pharmacy review and specialist medication pathways (anticoagulation, diabetes, resistant hypertension) were acknowledged, but not specified as core components. The model instead ensures that referrals to these services are triggered earlier and that discharge letters include medication actions and named services, leaving detailed optimization to local protocols. Because these pathways and their capacity vary by site, feasibility work will assess acceptability, adoption, fidelity, and resource impact, and adapt the pathway where capacity is constrained. Implementation will also vary with waiting-list length across procedures and with the temporal effects of post-COVID recovery. Hospitals with short waits may have little time to act, while those with longer waits may risk disengagement or inequity. These contextual factors will influence the fidelity and equity of delivery, and the authors will evaluate their impact during feasibility testing.

## Supplementary Material

znaf246_Supplementary_Data

## Data Availability

Data sharing requests will be considered by the writing group upon written request to the corresponding author.
