# Supplementary material for: Integrated care for people with multimorbidity into elective surgical pathways: mixed-methods co-design study
Source: Br J Surg. 2025 Nov 12;112(11):znaf246. doi: 10.1093/bjs/znaf246 (PMC12605797; doi:10.1093/bjs/znaf246)
Supplement: znaf246_Supplementary_Data [file znaf246_supplementary_data.docx]

**Integrated care for people with multimorbidity into elective surgical pathways: A mixed-methods co-design study**

Sivesh K Kamarajah^1, 2^, Jugdeep Dhesi^3, 4^, Kamlesh Khunti^5^, Krishnarajah Nirantharakumar^3^, Paul Cockwell^6^, Clare Hughes^6^, Paul Stern^7^, Joyce Yeung^8^, Dion G Morton^1, 2^, Aneel A Bhangu^†1, 2^, Shalini Ahuja^†9^

*^†^joint senior authors*

**Affiliations:**

1. Department of Applied Health Sciences, School of Health Sciences, College of Medicine and Health, University of Birmingham, Birmingham, UK.
2. NIHR Global Health Research Unit on Global Surgery, University of Birmingham, Birmingham, UK.
3. Department of Population Health Sciences, Faculty of Life Sciences and Medicine, Kings College London, London, UK.
4. Department of Health and Ageing, Guys and St Thomas' NHS Foundation Trust, London, UK.
5. Diabetes Research Centre, Leicester General Hospital, University of Leicester, Leicester, UK
6. University Hospital Birmingham NHS Trust, Birmingham, UK.
7. Head of Midlands Cardiovascular & Respiratory Clinical Network, NHS England.
8. Warwick Clinical Trials Unit, Warwick Medical School, University of Warwick, Coventry, UK.
9. Centre for Implementation Science, Health Services and Population Research Department, Institute of Psychiatry, Psychology and Neurosciences, King's College London, London, UK.

**Corresponding Author:** Mr Sivesh K Kamarajah, NIHR Doctoral Fellow, Department of Applied Health Sciences, School of Health Sciences, College of Medicine and Health, University of Birmingham, Birmingham, UK. **Email**: [s.k.kamarajah@bham.ac.uk](mailto:s.k.kamarajah@bham.ac.uk)

**Number of words:** 3,767 words **Number of pages:** 12

**Number of tables:** 1

**Number of figures:** 4

**Abstract word count:** 492 words

**Key words:** multiple long-term conditions; integrated care; complex intervention; implementation science; theory of change

**Short title:** Co-design for people with MLTC undergoing surgery

**Data sharing:** Data sharing requests will be considered by the writing group upon written request to the corresponding authors.

**Conflict of interest:** There are no conflicts of interest to declare.

**Supplementary Materials - Index**

| **Supplementary Methods** |  |  |  |
| --- | --- | --- | --- |
| TABLE S1. SUMMARY OF THE GUIDANCE FOR THE REPORTING OF INTERVENTION DEVELOPMENT (GUIDED) RECOMMENDATIONS. | | | *pag. 4* |
| TABLE S2. KEY DEFINITIONS WITHIN THE THEORY OF CHANGE | | | *pag. 6* |
| **Supplementary Results** | | |  |
| TABLE S3. SUMMARY OF THE EXPERT STAKEHOLDERS INVOLVED IN THE PATHWAY MAPPING PROCESS | | | *pag. 7* |
| TABLE S4. SUMMARY OF SCOPING REVIEW OF CLINICAL PRACTICE GUIDELINES PUBLISHED IN THE UNITED KINGDOM AROUND ADDRESSING PEOPLE WITH MULTIPLE LONG-TERM CONDITIONS | | | *pag. 8* |
| TABLE S5. SUMMARY OF THE KEY BARRIERS AND FACILITATORS FOR SMOKING CESSATION PATHWAYS | | | *pag.9* |
| TABLE S6. SUMMARY OF THE KEY BARRIERS AND FACILITATORS FOR ALCOHOL CESSATION PATHWAYS | | | *pag. 10* |
| TABLE S7. SUMMARY OF THE KEY BARRIERS AND FACILITATORS FOR WEIGHT MANAGEMENT PATHWAYS | | | *pag. 11* |
| TABLE S8. SUMMARY OF THE KEY BARRIERS AND FACILITATORS FOR SCREENING OF LONG-TERM CONDITIONS | | | *pag. 12* |
| TABLE S9. SUMMARY OF THE KEY BARRIERS AND FACILITATORS FOR MANAGEMENT OF LONG-TERM CONDITIONS | | | *pag. 13* |
| TABLE S10. SUMMARY OF THE KEY BARRIERS AND FACILITATORS FOR MEDICATION OPTIMISATION | | | *pag. 14* |
| TABLE S11. SUMMARY OF THE KEY BARRIERS AND FACILITATORS FOR MEDICATION OPTIMISATION | | | *pag. 15* |
| TABLE S12. SUMMARY OF COMPONENTS OF THE THEORY OF CHANGE | | | *pag. 16* |
| **Supplementary Appendixes** | | |  |
| APPENDIX A. NATIONAL SURVEY TO UNDERSTAND CURRENT PERIOPERATIVE PATHWAYS FOR PEOPLE WITH MULTIPLE LONG-TERM CONDITIONS | | | *pag. 17* |
| APPENDIX B. DETAILED DESCRIPTION OF THE THEORY OF CHANGE METHODOLOGY | | | *pag. 19* |
| APPENDIX C. DRAFT TOPIC GUIDE FOR THEORY OF CHANGE FOCUS GROUPS WITH HEALTHCARE PROFESSIONALS | | | *pag. 20* |
| **References** | | | *pag. 21* |

**Supplementary Methods**

## **Table S1.** Summary of the GUIDance for the rEporting of intervention Development (GUIDED) recommendations.

| Item description | Explanation | Page in manuscript where item is located |
| --- | --- | --- |
| Report the context for which the intervention was developed. | Understanding the context in which an intervention was developed informs readers about the suitability and transferability of the intervention to the context in which they are considering evaluating, adapting or using the intervention. Context here can include place, organisational and wider socio- political factors that may influence the development and/or delivery of the intervention (15). | 5 |
| Report the purpose of the intervention development process. | Clearly describing the purpose of the intervention specifies what it sets out to achieve. The purpose may be informed by research priorities, for example those identified in systematic reviews, evidence gaps set out in practice guidance such as The National Institute for Health and Care Excellence or specific prioritisation exercises such as those undertaken with patients and practitioners through the James Lind Alliance. | 5 |
| Report the target population for the intervention development process | The target population is the population that will potentially benefit from the intervention – this may include patients, clinicians, and/or members of the public. If the target population is clearly described then readers will be able to understand the relevance of the intervention to their own research or practice. Health inequalities, gender and ethnicity are features of the target population that may be relevant to intervention development processes. | 5-6 |
| Report how any published intervention development approach contributed to the development process | Many formal intervention development approaches exist and are used to guide the intervention development process (e.g. 6Squid (16) or The Person Based Approach to Intervention Development (17)). Where a formal intervention development approach is used, it is helpful to describe the process that was followed, including any deviations. More general approaches to intervention development also exist and have been categorised as follows  (3):- Target Population-centred intervention development; evidence and theory-based intervention development; partnership intervention development; implementation-based intervention development; efficacy- based intervention development; step or phased-based intervention development; and intervention-specific intervention development (3). These approaches do not always have specific guidance that describe their use. Nevertheless, it is helpful to give a rich description of how any published approach was operationalised | 6 |
| Report how evidence from different sources informed the intervention development process | Reporting whether and how theory informed the intervention development process aids the reader’s understanding of the theoretical rationale that underpins the intervention. Though not mentioned in the e-Delphi or consensus meeting, it became increasingly apparent through the development of our guidance that this theory item could relate to either existing published theory or programme theory. | 6-8 |
| Report how/if published theory informed the intervention development process. | Some interventions are developed with components that have been adopted from existing interventions. Clearly identifying components that have been adopted or adapted and acknowledging their original source helps the reader to understand and distinguish between the novel and adopted components of the new intervention. | 6 |
| Report any guiding principles, people or factors that were prioritised when making decisions during the intervention development process. | Reporting any guiding principles that governed the development of the application helps the reader to understand the authors’ reasoning behind the decisions that were made. These could include the examples of particular populations who views are being considered when designing the intervention, the modality that is viewed as being most appropriate, design features considered important for the target population, or the potential for the intervention to be scaled up. | 5-6 |
| Report how stakeholders contributed to the intervention development process | Potential stakeholders can include patient and community representatives, local and national policy makers, health care providers and those paying for or commissioning health care. Each of these groups may influence the intervention development process in different ways. Specifying how differing groups of stakeholders contributed to the intervention development process helps the reader to understand how stakeholders were involved and the degree of influence they had on the overall process. Further detail on how to integrate stakeholder contributions within intervention reporting are available (19). | 6-8 |
| Report how the intervention changed in content and format from the start of the intervention development process. | Intervention development is frequently an iterative process. The conclusion of the initial phase of intervention development does not necessarily mean that all uncertainties have been addressed. It is helpful to list remaining uncertainties such as the intervention intensity, mode of delivery, materials, procedures, or type of location that the intervention is most suitable for. This can guide other researchers to potential future areas of research and practitioners about uncertainties relevant to their healthcare context. | 6-8 |
| Report any changes to interventions required or likely to be required for subgroups. | Specifying any changes that the intervention development team perceive are required for the intervention to be delivered or tailored to specific sub groups enables readers to understand the applicability of the intervention to their target population or context. These changes could include changes to personnel delivering the intervention, to the content of the intervention, or to the mode of delivery of the intervention. | NA |
| Report important uncertainties at the end of the intervention development process. | Intervention development is frequently an iterative process. The conclusion of the initial phase of intervention development does not necessarily mean that all uncertainties have been addressed. It is helpful to list remaining uncertainties such as the intervention intensity, mode of delivery, materials, procedures, or type of location that the intervention is most suitable for. This can guide other researchers to potential future areas of research and practitioners about uncertainties relevant to their healthcare context. | 9-12 |
| Follow TIDieR guidance when describing the developed intervention. | Interventions have been poorly reported for a number of years. In response to this, internationally recognized guidance has been published to support the high quality reporting of health care? interventions^5^and public health interventions^14^. This guidance should therefore be followed when describing a developed intervention. | 21 |
| Report the intervention development process in an open access format. | Unless reports of intervention development are available people considering using an intervention cannot understand the process that was undertaken and make a judgement about its appropriateness to their context. It also limits cumulative learning about intervention development methodology and observed consequences at later evaluation, translation and implementation stages. Reporting intervention development in an open access (Gold or Green) publishing format increases the accessibility and visibility of intervention development research and makes it more likely to be read and used. Potential platforms for open access publication of intervention development include open access journal publications, freely accessible funder reports or a study web-page that details the intervention development process | 9-12 |

## **Table S2.** Key definitions within the theory of change

| **Terminology** | **Definition (adapted from De Silva, 2015)** |
| --- | --- |
| Impact | The real world impact we are trying to achieve for people with MLTC undergoing elective cancer surgery |
| Ceiling of accountability | The point at which we stop accepting responsibility for achieving those outcomes solely through the intervention programme. |
| Long-term outcomes | The outcome that the programme can achieve on its own. This can inspire  the choice for primary and secondary outcomes in the evaluation of the intervention. |
| Preconditions | A precondition or intermediate outcome is a necessary requirement, condition or element that needs to be realised for the desired outcome to be achieved. In the context of MLTC, these preconditions are the precursors or requirements for accomplishing successful integrated care in surgical pathways. |
| Intervention (actions or activities) | The different components of the complex intervention. They represent certain “actions” that need to be undertaken to bring about a certain result, intermediate outcome or precondition. These are “those things that the programme must do to bring about the outcomes”. |
| Assumptions | An external condition beyond the control of the project that must or is assumed to exist for the outcome to be achieved. |
| Rationales | The facts or reasons (based on evidence or experience) behind the choice of the intervention activities or strategies and each link of the causal pathway. |

# Phase 1A: Pathway mapping

## **Table S3**. Summary of the expert stakeholders involved in the pathway mapping process

| **Surgery** |  |
| --- | --- |
| Prof Dion Morton | Professor of Surgery, University of Birmingham |
| Prof Aneel Bhangu | Professor of Global Surgery, University of Birmingham |
| Prof Thomas Pinkney | Professor of Surgical Trials, University of Birmingham; NIHR CRN Lead for Surgery |
| Mr Mark Cheetham | Consultant Surgeon, Shrewsbury NHS Trusts; GIRFT Surgical Lead |
| **Perioperative Care** |  |
| Dr Adam Low | Consultant Neuroanaesthetist, Clinical Service Lead for Pre Assessment & Perioperative Care, University Hospital Birmingham NHS Trust |
| Prof Joyce Yeung | Professor of Perioperative Care; NIHR CRN Lead for Perioperative Care; Perioperative Clinical Trials Network Chair |
| Dr. Michael Swart | GIRFT Perioperative Lead |
| **Geriatric medicine** |  |
| Prof. Jugdeep Dhesi | Professor Geriatric Medicine, King’s College London; Co-Director, Centre for Perioperative Care |
| Prof. Miles Witham | Professor of Geriatric Medicine, Newcastle University |
| Dr. Mark Johnston | Consultant Geriatrician, Liverpool Hospitals NHS Trust |
| Dr. Clare Hughes | Consultant Geriatrician, University Hospital Birmingham NHS Trust |
| **Public Health** |  |
| Prof. Krishnarajah Nirantharakumar | Professor of Public Health, University of Birmingham |
| Mr Paul Stern | Head of Network for Long Term Conditions and Prevention, NHS England Midlands |
| **Health system research** |  |
| Dr Shalini Ahuja | Lecturer in Health Systems Research |
| **Primary care** |  |
| Prof. Clare Taylor | Professor of Primary Care, University of Birmingham |
| **Patient and public member** |  |
| Sue Blackwell | Patient representative, Liverpool |
| **Allied Health Professionals** |  |
| Ms. Emma McCone | Getting It Right First Time National lead for Preoperative Assessment, NHS England |
| Ms. Christina Reihill | Preoperative Assessment Lead, University Hospital Birmingham NHS Trust |
| **Integrated care board** |  |
| Paul Cockwell | Consultant Nephrologist, University Hospital Birmingham NHS Trust Professor of Nephrology, University of Birmingham  Medical Director Long Term Conditions and Prevention Birmingham and Solihull |
| Sudhakar Shinde | Head of Prevention and Long Term Conditions, NHS Birmingham and Solihull |
| Clara Day | Chief Medical Officer NHS Birmingham and Solihull |

**Supplementary Results**

# Phase 1B: Policy and clinical practice guideline scan

## **Table S4.** Summary of policy and clinical practice guidelines published in the United Kingdom around addressing people with multiple long-term conditions.

| **Domain** | **Recommendation** | **Source** |  |
| --- | --- | --- | --- |
| **Multiple long-term conditions** | | | |
| Screening | Early screening for perioperative risk factors at the point of referral or listing | NHS England 2023 |  |
|  | Use of validated risk stratification tool for risk assessment | NICE NG180 |  |
|  | Perform frailty assessment | Keller et al 2024 |  |
| Model of care | Involve specialist teams (cardiology, endocrinology, geriatricians, etc.) early for complex comorbidities. | CPOC 2021,  NHS England 2023 |  |
| **Diabetes** | | |  |
| Screening | Measure HbA1c ideally within 3 months. If >69 mmol/mol (~8.5%), consider deferring elective surgery until better control, balancing urgency. | CPOC-Diabetes UK 2023 |  |
| Management | Improve glycaemic control pre-op; aim for HbA1c <69 mmol/mol if feasible. Ensure comorbidities (renal, cardiac) and diabetic complications are addressed (foot checks, neuropathy). | CPOC-Diabetes UK 2023 |  |
|  | Minimise fasting; continue basal insulin in Type 1 diabetics. Consider a variable rate intravenous insulin infusion (VRIII) for moderate-major surgery or if poor control. Target intra-op glucose ~6 - 10 mmol/L. | CPOC-Diabetes UK 2023, NICE NG180 |  |
|  | Frequent capillary blood glucose checks; resume usual diabetes regimen early once eating/drinking. Overlap IV insulin with subcutaneous insulin to avoid ketoacidosis in Type 1 patients. | CPOC-Diabetes UK 2023 |  |
| **Hypertension** | | |  |
| Screening | Check BP at pre-op clinic. If BP ≥180/110 mmHg, consider deferring elective surgery until better controlled. Target <160/100 mmHg if possible. | NICE NG180 |  |
| Management | Continue antihypertensives (beta-blockers, CCBs, diuretics) up to the day of surgery. Hold ACE inhibitors/ARBs on morning of surgery to reduce intra-op hypotension risk (case by case). | CPOC (2021) |  |
| **Smoking** | | |  |
| Management | Advise smoking cessation as early as possible (≥4 weeks pre-surgery). Provide cessation support (nicotine replacement, referral). | NICE NG180; CPOC (2021) |  |
| **Alcohol** | | |  |
| Screening | Screen for hazardous drinking. Recommend abstinence/reduction for ≥4 weeks before surgery. | NICE NG180; Cochrane Reviews |  |
| Management | Provide support for alcohol dependence if needed. |  |  |
| **Weight management** | | |  |
| Screening | Nutritional screening for all major surgery patients; address malnutrition or obesity. | CPOC 2021; SAGES/EAES 2024 |  |
| Management | Consider prehabilitation for weight optimisation, muscle strengthening, and improved cardio-respiratory fitness. |  |  |

# Phase 1C: National survey of practice

## **Table S5.** Summary of the key barriers and facilitators for smoking cessation pathways

| Construct | Theme | Type | Frequency, n |
| --- | --- | --- | --- |
| Inner Setting |  |  |  |
| Available Resources | Lack of funding, time, staff, or service capacity | Barrier | 8 |
| Structural Characteristics | Pre-op pathway too short; limited integration in small NHS boards | Barrier | 1 |
| Implementation Climate | Smoking not prioritised, no ownership, unclear cultural norms | Barrier | 2 |
| Outer Setting |  |  |  |
| Patient Needs & Resources | Socioeconomic barriers, postcode lottery, patient stress due to cancer diagnosis | Barrier | 4 |
| Cosmopolitanism | Over-reliance on GPs, lack of communication across sectors | Barrier | 4 |
| External Policy & Incentives | No secondary care funding; policy decisions preventing action | Barrier | 3 |
| Characteristics of Individuals |  |  |  |
| Knowledge & Beliefs | Clinician awareness or misunderstanding of smoking cessation pathways | Barrier | 3 |
|  | Empowered clinicians promoting cessation | Facilitator | 5 |
| Self-Efficacy | Low confidence among staff on how/when to refer | Barrier | 2 |
| Individual Stage of Change | Patient motivation to quit varies; staff perseverance | Barrier | 2 |
|  | Motivated patients and responsive clinicians | Facilitator | 6 |
| Intervention Characteristics |  |  |  |
| Design Quality & Packaging | No clear or standardised protocols; inconsistent patient materials | Barrier | 5 |
|  | Posters, clear written pathways, structured processes | Facilitator | 10 |
| Adaptability | Online referrals, electronic records reduce administrative burden | Facilitator | 4 |
| Process |  |  |  |
| Planning | Lack of embedded planning or referral points across the care journey | Barrier | 3 |
| Engaging | Staff proactively identify smokers, offer counselling, refer at multiple points | Facilitator | 7 |

## **Table S6.** Summary of the key barriers and facilitators for alcohol cessation pathways

| Construct | Theme | Type | Frequency, n |
| --- | --- | --- | --- |
| Inner Setting |  |  |  |
| Available Resources | Lack of funding, understaffing, no dedicated alcohol services | Barrier | 8 |
| Structural Characteristics | Short time to surgery, especially in cancer; lack of formal pathways | Barrier | 4 |
| Outer Setting |  |  |  |
| Patient Needs & Resources | Alcohol dependence under-recognised; postcode-based variability in referral options | Barrier | 3 |
| Cosmopolitanism | Over-reliance on GPs; unclear interface between primary and secondary care | Barrier | 3 |
| Characteristics of Individuals |  |  |  |
| Knowledge & Beliefs | Staff awareness variable; some proactive awareness-raising via education | Mixed | 3 (2 barriers; 1 facilitator) |
| Self-Efficacy | Some staff lack confidence in screening, advising, or referring for alcohol issues | Barrier | 2 |
| Individual Stage of Change | Some patients unmotivated or disengaged; others benefit from ‘teachable moments’ | Mixed | 3 (2 barriers; 1 facilitator) |
| Intervention Characteristics |  |  |  |
| Design Quality & Packaging | Ad hoc or missing referral processes; some mention of protocols, posters, and screening prompts | Mixed | 4 (2 barriers; 2 facilitator) |
| Adaptability | Online or portal-based referrals are time-saving and easier to implement | Facilitator | 1 |
| Process |  |  |  |
| Planning | No written protocols; services added late in pathway; lack of structured approach | Barrier | 2 |
| Engaging | Pre-op nurses, CNS, and prehab teams actively screen and refer where resources exist | Facilitator | 2 |

## **Table S7.** Summary of the key barriers and facilitators for weight management pathways

| Construct | Theme | Type | Frequency, n |
| --- | --- | --- | --- |
| Inner Setting |  |  |  |
| Available Resources | Funding gaps, limited staffing, space constraints, lack of physiotherapy capacity | Barrier | 17 |
| Structural Characteristics | Short interval from decision to surgery, small NHS boards, hospital logistics | Barrier | 3 |
| Outer Setting |  |  |  |
| External Policy & Incentives | Access limited to cancer cases; variation in provision by trust or region | Barrier | 2 |
| Characteristics of Individuals |  |  |  |
| Knowledge & Beliefs | Awareness varies among staff; poor promotion to clinicians/patients | Barrier | 1 |
| Self-Efficacy | Staff unsure how or when to refer; lack of training or confidence | Facilitator | 1 |
| Individual Stage of Change | Patients engaged and motivated to participate in improving health | Facilitator | 1 |
| Intervention Characteristics |  |  |  |
| Design Quality & Packaging | No structured pathway; reliant on research legacy, informal advice, or referrals | Barrier | 2 |
| Process |  |  |  |
| Planning | No checklist; poor integration with decision points | Barrier | 1 |
| Engaging | Active CNS/physio/anaesthetic teams driving engagement (but limited by service priorities) | Mixed | 2 (1 barriers; 1 facilitator) |

## **Table S8.** Summary of the key barriers and facilitators for screening of long-term conditions

| Construct | Theme | Barriers | Facilitators |
| --- | --- | --- | --- |
| Inner Setting |  |  |  |
| Networks & Communication | Internal coordination and handovers | Delays in response from other services; unclear handover to GPs or specialists | Multidisciplinary team (MDT) models; coordinated perioperative medicine (POM) services |
| Culture | Ownership and engagement | Screening viewed as outside remit of surgical or pre-op teams | Willingness of staff; protocols empowering nurses and anaesthetists |
| Implementation Climate | Organisational readiness | IT insufficiencies, staffing shortages, lack of joined-up thinking | Dedicated dashboards, structured workflows, better referral tracking |
| Outer Setting |  |  |  |
| Patient Needs & Resources | Interface with primary care | Poor communication, unclear responsibilities for follow-up | Seamless primary-secondary care integration; GP optimisation support |
| External Policy & Incentives | Commissioning and resource prioritisation | Lack of funding, inadequate commissioning, and low priority from leadership | Business cases to commissioners; POPS implementation; prehabilitation teams |
| Characteristics of Individuals |  |  |  |
| Knowledge & Beliefs | Clinician attitudes towards screening | Ambiguity over who takes responsibility; awareness of optimisation benefits varies | Strong belief in value of early identification and intervention |
| Intervention Characteristics |  |  |  |
| Complexity | Complexity of pathways | Pathways are inconsistent, ad hoc, or unclear; lack of standardisation | Clear referral protocols; dashboards to identify risk factors; nurse-led preop screening |
| Adaptability | Timing in the surgical pathway | Screening done too late (e.g. 2–3 weeks pre-op) for optimisation | Early screening via electronic questionnaires or at time of listing |
| Process |  |  |  |
| Engaging | Engaging key stakeholders | Surgeons, nurses, and specialists not uniformly engaged or informed | Regular training, visual pathways (e.g., posters), clear role assignments |
| Executing | Operational delivery of screening | Limited personnel, time constraints, lack of protocol-driven approach | Nurse-led or automated screening systems; POPS clinics; CGA pathways |

## **Table S9.** Summary of the key barriers and facilitators for management of long-term conditions

A CFIR-based thematic analysis revealed significant variation in the optimisation of chronic conditions before surgery. While established pathways exist for diabetes, anaemia, and anticoagulation, approaches for frailty and cardiopulmonary disease remain inconsistent and often improvised. Late referrals typically occurring just 2-3 weeks before surgery, limit opportunities for effective intervention. Key barriers include unclear referral responsibilities, weak integration with primary care, workforce shortages, and insufficient commissioning of perioperative services. Facilitators include dedicated perioperative teams, reflex referral protocols, multidisciplinary clinics (e.g. Perioperative care of Older People undergoing Surgery, Comprehensive Geriatric Assessment), and structured pre-assessment pathways. Improving optimisation and perioperative outcomes requires strong leadership, early identification, and enhanced cross-sector communication.

| **Construct** | **Theme** | **Barriers** | **Facilitators** |
| --- | --- | --- | --- |
| **Inner Setting** |  |  |  |
| Networks & Communication | Internal coordination and handovers | Delays in response from other services; unclear handover to GPs or specialists | Multidisciplinary team (MDT) models; coordinated perioperative medicine (POM) services |
| Culture | Ownership and engagement | Screening viewed as outside remit of surgical or pre-op teams | Willingness of staff; protocols empowering nurses and anaesthetists |
| Implementation Climate | Organisational readiness | IT insufficiencies, staffing shortages, lack of joined-up thinking | Dedicated dashboards, structured workflows, better referral tracking |
| **Outer Setting** |  |  |  |
| Patient Needs & Resources | Interface with primary care | Poor communication, unclear responsibilities for follow-up | Seamless primary-secondary care integration; GP optimisation support |
| External Policy & Incentives | Commissioning and resource prioritisation | Lack of funding, inadequate commissioning, and low priority from leadership | Business cases to commissioners; POPS implementation; prehabilitation teams |
| **Characteristics of Individuals** |  |  |  |
| Knowledge & Beliefs | Clinician attitudes towards screening | Ambiguity over who takes responsibility; awareness of optimisation benefits varies | Strong belief in value of early identification and intervention |
| **Intervention Characteristics** |  |  |  |
| Complexity | Complexity of pathways | Pathways are inconsistent, ad hoc, or unclear; lack of standardisation | Clear referral protocols; dashboards to identify risk factors; nurse-led preop screening |
| Adaptability | Timing in the surgical pathway | Screening done too late (e.g. 2–3 weeks pre-op) for optimisation | Early screening via electronic questionnaires or at time of listing |
| **Process** |  |  |  |
| Engaging | Engaging key stakeholders | Surgeons, nurses, and specialists not uniformly engaged or informed | Regular training, visual pathways (e.g., posters), clear role assignments |
| Executing | Operational delivery of screening | Limited personnel, time constraints, lack of protocol-driven approach | Nurse-led or automated screening systems; POPS clinics; CGA pathways |

.

## **Table S10.** Summary of the key barriers and facilitators for medication optimisation

Medication management and optimisation during the perioperative period varies significantly and is often under-resourced across services. While high-risk medications such as anticoagulants, antihypertensives, and diabetes therapies, receive consistent attention, comprehensive medication reviews, addressing polypharmacy and appropriateness are infrequent. In many cases, reviews are confined to late-stage pre-assessment or conducted inconsistently based on clinician availability. Key challenges include a shortage dedicated perioperative pharmacists, limited time in outpatient clinics, unclear responsibility across anaesthetic, surgical, and pharmacy teams, and inadequate commissioning. However, effective facilitators include nurse-led protocols, pharmacist-supported anaesthetic clinics, and comprehensive geriatric assessment (CGA) or POPS services that integrate medication reviews into MLTC management. Institutions with embedded pharmacy support report greater confidence in safe perioperative prescribing and deprescribing. Expanding structured medication optimisation pathways, starting as early as referral or diagnosis, could substantially enhance patient safety and surgical preparedness.

| **Construct** | **Theme** | **Barriers** | **Facilitators** |
| --- | --- | --- | --- |
| **Inner Setting** |  |  |  |
| Networks & Communication | Interdisciplinary coordination | Clinicians report limited input from pharmacy or COE; unclear responsibility for optimisation | Nurse-led pre-op clinics issuing tailored advice; anaesthetic/pharmacy flagging systems |
| Implementation Climate | Role clarity and readiness | Pharmacy, anaesthetics, and surgery vary in perception of responsibility | Defined role for POPS or CGA teams; central coordination (e.g. POM nurse) |
| **Outer Setting** |  |  |  |
| Patient Needs & Resources | Pharmacy resource availability | Widespread lack of dedicated perioperative pharmacists; pharmacy sees it as outside their remit | Business cases for peri-op pharmacists; MDT collaboration (e.g. with DSNs, geriatricians) |
| External Policy & Incentives | Funding and commissioning | No funding allocated to perioperative pharmacy services; service development deprioritised | Primary care reviews at referral; cross-boundary pathways (e.g. rapid diagnostic centres) |
| **Characteristics of Individuals** |  |  |  |
| Knowledge & Beliefs | Staff capacity and confidence | Time pressure; concern that full medication review is too complex | Clinician willingness where capacity exists; local champions (e.g. pharmacists or anaesthetists) |
| **Intervention Characteristics** |  |  |  |
| Complexity | Scope and consistency of medication review | Medication optimisation often limited to high-risk drugs (e.g. anticoagulants); full medication review not routinely done | Standardised templates; defined protocols for key drug classes; integrated medication checklists |
| Adaptability | Timing of review | Medication review occurs late (2–3 weeks pre-op) or only on day of surgery listing | Early pharmacist/POPS input; virtual reviews; integration into prehabilitation |
| **Process** |  |  |  |
| Engaging | Pharmacy engagement and training | Poor pharmacy involvement; variable training and awareness | Posters, training sessions; designated clinics with pharmacist access |
| Executing | Execution of routine medication review | Inconsistent or “hit and miss” reviews; lack of pathways for polypharmacy | POPS and CGA clinics; nurse/pharmacist-led services; automated flags in assessment tools |

## **Table S11.** Summary of the key barriers and facilitators for medication optimisation

Perioperative care planning is a vital aspect of patient-centred surgical care, yet its implementation remains inconsistentacross healthcare settings. Specialist nurses typically lead this process, offering patients with personalised information, recovery expectations, and key contact points. However, in environments with limited CNS staffing, care plans are often delayed, generic, or fragmented across teams. Anaesthetists and surgeons frequently express uncertainty regarding their roles and the scope of care planning. Barriers include workforce shortages, time constraints, and insufficient IT and administrative support for coordinating materials and follow-up. Effective strategies include embedded ERAS protocols, integration of prehabilitation and surgical school resources, and the use of structured information booklets or apps. When executed well, care plans enhance patient understanding, satisfaction, and preparedness for surgery. Strengthening interprofessional coordination, expanding CNS capacity, and standardising care plan content across services are crucial for improving implementation fidelity and ensuring equitable access.

| **Construct** | **Theme** | **Barriers** | **Facilitators** |
| --- | --- | --- | --- |
| **Inner Setting** |  |  |  |
| Implementation Climate | Readiness for implementation | Some services lack ERAS integration or administrative infrastructure | Strong collaboration between admin and clinical teams; established POM clinics |
| Networks & Communication | Communication within and across teams | Disjointed input between pre-op, CNS, and surgical teams | Centralised preassessment hubs; MDT communication; care summaries coordinated across services |
| **Outer Setting** |  |  |  |
| Patient Needs & Resources | Personalisation and engagement | Patients appreciate tailored info but often receive fragmented input from multiple professionals | Individualised ERAS plans; holistic assessments; provision of contact points (CNS, admin) |
| External Policy & Incentives | Funding and service models | CNS staffing shortages; lack of time and protected resource; variable pathway access | Business cases submitted; ERAS embedded in many surgical services; well-defined local protocols |
| **Characteristics of Individuals** |  |  |  |
| Knowledge & Beliefs | Clinician understanding of care planning | Some clinicians unclear about what constitutes a care plan; uncertainty about whose role it is | Champions within nursing and anaesthetic teams promote patient engagement and consistency |
| **Intervention Characteristics** |  |  |  |
| Design Quality & Packaging | Format and accessibility of care plans | Inconsistent documentation; absence of centralised booklets; variable use of digital tools | Use of third-party apps, printed care diaries, personalised letters, and condition-specific leaflets |
| Complexity | Role distribution and pathway clarity | Care plans depend on CNS availability; surgeons and pre-op teams report unclear ownership | Nurse-led education sessions; prehabilitation clinics; structured pre-op appointments |
| **Process** |  |  |  |
| Executing | Delivery of care plan | Time constraints, remote assessments, and stretched staff delay care plan creation | Early pre-op appointments, stoma education, rehab plans and CGA inputs standardised in some settings |
| Engaging | Engaging patients and families | Limited time to support patients holistically; variable follow-up | Prehabilitation signposting, written guidance, enhanced recovery education, family involvement |

# Phase 2: Iterative development of Theory of Change

## **Table S12.** Summary of components of the theory of change

| **Level** | **Key components of the theory of change** |
| --- | --- |
| **Definitions** | **Healthy longevity:** functional independence (EQ-5D mobility ≥ baseline) and no MLTC-related readmissions over 12 months.  **MLTC-related complications:** postoperative events driven by uncontrolled diabetes, hypertension, or smoking (e.g., hyper/hypoglycaemia, hypertensive crisis, pneumonia). |
| **Goal / impact** | Extend healthy longevity after elective surgery by reducing MLTC-related complications and preserving functional independence. |
| **Long-term outcomes**  (12 m after surgery) | - ≥15-20% improvement in *Composite Health Score* (EQ-5D-5L) - ≥20% absolute increase in sustained control of diabetes (HbA1c < 58 mmol/mol) and hypertension (< 140/90 mm Hg) in GP records. |
| **Intermediate outcomes**  (30 d → 6 m postop) | - ≥30 % fewer MLTC-related readmissions - ≥15 % shorter median LOS for high-risk patients - ≥80 % of GPs document action on optimisation plan within 3 months. |
| **Short-term outcomes**  (Surgery Day 0 → 30 d) | - ≥15-20 % fewer Clavien-Dindo ≥ II medical complications - ≥ 20 % fewer Day-0 cancellations due to uncontrolled diabetes, hypertension or ongoing smoking. |
| **Immediate outcomes** (*Day L listing → Day L + 14*) | - ≥ 95 % of listed patients have a completed *Health-Check Checklist* (diabetes, BP, smoking) - ≥ 90 % of positive screens auto-refer to GP or disease-specific nurse within 48 hours - ≥ 75 % of flagged patients start optimisation (med-titration, cessation booking) within 2 weeks of listing. |
| **Preconditions** | - Checklist embedded in e-Referral & surgical clinic EPR - Auto-alerts route positive screens to GP task list / specialist nurse - Standard operating protocol: surgeon or clinic nurse completes checklist at listing; GP acts - Patient receives brief advice + QR links - Monthly dashboard to surgical team (completion & optimisation KPIs). |
| **Intervention (strategies)** | - A healthcare checklist - Early identification of patients living with MLTC - Patient activation materials - Use of the perioperative window to ensure optimisation of MLTC - Structured discharge communication. |
| **Indicators** | - Proportion of completion and accuracy of checklist - Cancellation of surgery - 30-day complications - 30-day readmission - Composite Health Score - Controlled HbA1c or blood pressure at 12 months |
| **Ceiling of accountability** | - Funding and costs to develop sustainable pathways - Long-term control relies on GP follow-through and patient self-management. |
| **Rationale** | - Moving screening to **Day L** creates ≥ 6 week optimisation window (average UK wait 8-12 weeks) - Rapid GP or specialist engagement improves control before the metabolic stress of surgery - Structured hand-off & GP prompt keep momentum after discharge, reducing relapse - Cumulative gains leads to fewer peri-op setbacks, faster recovery and maintained independence. |
| **Assumptions** | - Each hospitals have good communication systems between primary care - Capacity within primary care to act on alerts - Access to smoking-cessation & chronic-disease services - Digital-exclusion mitigations in place. |

**Supplementary Appendixes**

## **Appendix A.** Patient and public involvement in the mixed-methods study Patient partners shaped the intervention in four concrete ways (Table Sx):

1. **Prioritisation of domains:** During the prioritisation exercise, patient partners argued for including **smoking** alongside **diabetes** and **hypertension**, emphasising self-management burden, access to support during long waits, and potential for quick gains. This input informed the final selection of the three target domains.
2. **Move upstream and automate handovers:** Patients described listing as the moment when “things start moving” but also when information is most likely to be missed. This led us to specify **checklist-based screening at listing** and **automated referrals** (rather than ad hoc) to reduce the risk of falling through gaps.
3. **Discharge continuity:** Patients requested that discharge letters include a **“What happens next”** section with actions, targets, timelines, and a named service. We therefore added **structured discharge communication** with explicit MLTC actions and responsible owners.

### **Table S13. Patient contributions and material changes to the intervention**

| **Patient input** | **Resulting change** | **Influence in study** |
| --- | --- | --- |
| Include smoking support with medical conditions; long waits are a “window to act” | Selected **diabetes, hypertension, smoking** as the three domains | Phase II |
| Listing is when mistakes happen; referrals must not rely on memory | Added **checklist at listing** and **automated e-referrals** | Table 2; Figure 2 |
| Tell me exactly what happens after discharge and who is responsible | Added **structured discharge communication** with actions/targets and named service | Table 2; discharge section |
| Make outcomes meaningful to patients (function and daily life) | Retained **EQ-5D-5L** at 12 months and linked intermediate milestones in ToC | Figure 4; Phase 2 |

## **Appendix B.** National survey to understand current perioperative pathways for people with multiple long-term conditions

| **Respondent characteristics** | **Required data (definition / comment)** |
| --- | --- |
| 1. **Name** | (name of respondent) |
| 1. **Role** | Surgeon / Anaesthetists / Nurse specialist (Cancer / Pre-operative / Other) / Critical care / Geriatrician / Allied Health Professional (Physiotherapist) |
| 1. **Email** | (email of respondent) |
| 1. **If interested, would you like to be contacted for a future focus group to explore pathway improvement for people with MLTC?** | No / Yes |
|  |  |
| **Current pathways** | **Required data (definition / comment)** |
| 1. **What model of care do you have for surgical patients before surgery? (tick all that apply)** | **None / Pre-operative assessment clinics** (Nurse-led / Consultant-led) / **Dedicated multidisciplinary clinics / Prehabilitation / Other** (please describe)  ***If pre-operative assessment clinics / Dedicated MDT Clinics:***   - *Which specialty members are part of the teams? (Surgeon / Anaesthesists / Critical care / Geriatrician / Pharmacists / Physiotherapists / Dietcians / Specialist nurses)* - *How are findings communicated to primary care? (Not communicated / Communicate via letter to GP / Communicate to a dedicated or named member in primary care)* |
| 1. **Do you have dedicated pathways or protocols for people with MLTC undergoing elective cancer surgery?** | **No / Yes** *(If yes, what is the pathway or protocol? Please upload if you have a copy of this)* |
|  |  |
| **Key interventions** | **Required data (definition / comment)** |
| **These key behaviours were identified by a systematic review delivered within different models of care for people with MLTC.** | |
| **Smoking cessation** | |
| 1. **Is smoking cessation addressed in the current surgical pathway?** | **No / Yes, sometimes / Yes, always**  *If yes:*   - *Who normally does this? (Surgical team member / Existing pathway)* - *Who should normally try and promote smoking cessation?* - *When does this normally occur?* |
| 1. **Are you aware of any existing pathways or support in your hospital?** | **No / Yes**  *If yes:*   - *Please describe this pathway* - *Do you face any issues or barriers within current pathways for these conditions?* - *What has worked well for these current pathways?* |
| **Alcohol consumption** |  |
| 1. **Is alcohol consumption of the patient addressed in the current surgical pathway?** | **No / Yes, sometimes / Yes, always**  *If yes:*   - *Who normally does this? (Surgical team member / Existing pathway)* - *Who should normally try and promote smoking cessation?*   *When does this normally occur?* |
| 1. **Are you aware of any existing pathways or support in your hospital?** | **No / Yes**  *If yes:*   - *Please describe this pathway* - *Do you face any issues or barriers within current pathways for these conditions?* - *What has worked well for these current pathways?* |
| **Physical activity** |  |
| 1. **Is physical activity addressed in the current surgical pathway?** | **No / Yes, sometimes / Yes, always**  *If yes:*   - *Who normally does this? (Surgical team member / Existing pathway)* - *Who should normally try and promote this?* - *When does this normally occur?* |
| 1. **Are you aware of any existing pathways or support in your hospital?** | **No / Yes**  *If yes:*   - *Please describe this pathway* - *Do you face any issues or barriers within current pathways for these conditions?* - *What has worked well for these current pathways?* |
| **Screening of long-term conditions.** This refers to the routine assessment of long-term conditions such as diabetes (blood glucose) and hypertension (blood pressure) | |
| 1. **Does routine screening of long-term conditions occur in the surgical pathway** | **No / Yes, sometimes / Yes, always**  *If yes:*   - *Who normally does this?* - *Which of the following long-term conditions are normally assessed? (Diabetes / Hypertension / Chronic kidney disease / Hyperlipidaemia)* |
| 1. **Once measurements for these conditions are taken, is there a pathway for diagnosis?** | **No / Yes - letter to GP / Yes - letter to relevant specialty / Other: please describe**  *If yes:*   - *Do you face any issues or barriers within current pathways for these conditions?* - *What has worked well for these current pathways?* |
| **Management of chronic diseases** |  |
| 1. **Are there structured referral pathways to integrate management of the following chronic conditions?** | **Diabetes / Hypertension / Anaemia / Chronic kidney disease**  *If yes to any:*  *(i) please describe how this occurs or upload a protocol.*  *(ii) Do you face any issues or barriers within current pathways for these conditions?*  *(iii) What has worked well for these current pathways?* |
| **Medication review and optimisation** |  |
| 1. **Is there a structured medication review and optimisation in the current surgical pathway?** | **No / Yes - selected patients / Yes - all patients**  *If yes to any:*   - *Who normally does this?* |
| **Other key interventions** |  |
| 1. **Do patients with MLTC receive detailed care plans?** | **No / Yes - sometimes / Yes - always**  *If yes to any:*   - *Who normally provides this?* - *Are these communicated to primary care teams? (Not communicated / Communicate via letter to GP / Communicate to a dedicated or named member in primary care)* |
| 1. **Is there a structured care coordination for people with MLTC in current surgical pathways?** | **No / Yes - sometimes / Yes - always**  *If yes to any:*   - *Who normally provides this?* - *Do you face any issues or barriers within current pathways for these conditions?* - *What has worked well for these current pathways?* - *Are these communicated to primary care teams? (Not communicated / Communicate via letter to GP / Communicate to a dedicated or named member in primary care)* |

## **Appendix C.** Detailed description of the Theory of Change methodology

A theory of change (ToC) is “a theory of how and why an initiative works which can be empirically tested by measuring indicators for every expected step on the hypothesised causal pathway to impact”.^1^ This is represented visually in a ‘ToC map’, offering a comprehensive depiction of how long-term outcomes can be achieved within a specific context and under defined conditions.^1^ To construct the map, the specific terms used are outlined in Table 1. The methodology behind ToC mapping, follows a process known as “backwards outcome mapping”, beginning with the definition of the desired ultimate impact and long-term outcomes. By systematically working backwards, all necessary intermediate outcomes or “pre- conditions” required to reach this envisioned goal are identified. This differs from the conventional “so-that” reasoning, facilitating deeper reflection on how the intervention will drive meaningful change.^1^

Integrating ToC principles into the NIHR / MRC framework has the potential to strengthen all four phases: (i) development; (ii) feasibility or piloting; (iii) evaluation; and (iv) implementation. During the development phase, a ToC approach can enhance stakeholder engagement, refine intervention design and ensure contextual relevance. In feasibility and pilot testing, it helps identify implementation barriers and assesses tje acceptability and applicability of the intervention. In the evaluation phase, the ToC map provides a structured approach for assessing implementation process, dinstinguising the critical factors that drive effectiveness.^1^ Based on implementation insights and evaluation evidence, the map can be refined to illustrate a ‘story’ of how integrated care pathways functioned within a specific setting.^1^

Stakeholders in this processed are individuals involved in the development, implementation or administration of surgical pathways. To construct the ToC and design the intervention, a diverse range of stakeholders was identified (Table S1). Initially, a Study Management Group was established, comprising experts with extensive national expertise in surgical and perioperative research and policy. Stakeholders were then purposively sampled and recruited through various channels, including social media promotions, word of mouth, and direct outreach by the authors within their professional networks. Recruitment was carried out by SKK, SA, AB and DGM via e-mails and follow-up telephone discussions, leveraging prior connections and the research group’s established expertise in surgery and perioperative care.

## **Appendix D.** Draft topic guide for theory of change focus groups with healthcare professionals

| **Key sections** | **Description** | **Duration** |
| --- | --- | --- |
| **Workshop 1 & 2** | | |
| 1. Welcome slides | Welcome everyone and introduce themselves. Then point out how this session via Zoom can work better if you:   - Mute if you are not talking. - Use the chat box to share thoughts as well if you wish. - Feel free to switch camera off - Use the hand up button | 7-8 minutes |
| 1. Brief overview | Give a brief overview of the study as a whole followed by the appropriate ‘housekeeping’ information.  Highlight the role of the group in the development of the intervention. Also point out that this will be a very interactive process, feel free to share your opinion whilst also respecting others which may differ to yours. | 2-3 minutes |
| 1. Moonshot | In an ideal world, what would need to happen for a successful implementation of integrated surgical pathways? | 10 minutes |
| **Workshop 1** | | |
| 1. Impact | Discuss on what the impact might look like: What is the fundamental change we want to see in secondary care systems for people with MLTC | 10 minutes |
| 1. Ceiling of accountability |  | 5 minutes |
| 1. Long-term outcomes | What would be the immediate long-term outcome we wish to achieve in the current surgical pathway? | 10 minutes |
| 1. Precondition | What are the intermediate preconditions that are necessary to  produce the long-term outcomes? Why do we think a given precondition will lead to (or is necessary to) reach the one that follows it? | 10 minutes |
|  | What contextual conditions or circumstances are necessary to achieve the preconditions? | 10 minutes |
| **Workshop 2** | | |
| 1. Refresh of ToC | Presentation and discussion of the ToC map developed In workshop 1 | 5 minutes |
| 1. Refining ToC | Review and refinement of the ToC developed in workshop 1 and filling in the gaps: Is the ToC map presented here “feasible” (likely to work), “effective” and “sustainable”? Is the change logically displayed? Are there essential elements that are missing or that we should definitely consider or discuss? | 10 minutes |
| 1. Interventions and strategies | Which interventions should be initiated to achieve the preconditions and the long-term outcome? | 30 - 45 minutes |
| **Workshop 1 & 2** | | |
| 1. Next steps | Summarise what has been achieved and mention when the next meeting is and its aim.  We should also stress the importance of contacting us if they wish to - email & telephone.  Keep meeting open if anyone wants to stay on to discuss any of the points raised - up to 90 minutes. Feel free to leave, but we are happy to hang around for a while and talk about anything really. | 5 minutes |

# Reference

1. De Silva MJ, Breuer E, Lee L, Asher L, Chowdhary N, Lund C, Patel V. Theory of Change: a theory-driven approach to enhance the Medical Research Council's framework for complex interventions. *Trials* 2014;**15**: 267.
